# Notes and News

**Published:** 1899-09-09

**Authors:** 


### NOTES AND NEWS.

(Collected and Communicated.)

According to the *Medical Record* of New York a law has recently been passed by the Michigan Legislature, forbidding the marriage of any person suffering from gonorrhœa or syphilis, and providing that anyone infringing this law shall be guilty of a felony punishable by fine or imprisonment, or by both, the imprisonment not to exceed five years. We venture, however, to doubt the advantage of passing laws of this kind which are sure to be broken, and are likely to be largely used as engines for the levying of blackmail.

How to do good without doing evil is a constant difficulty to the philanthropist. The Whitechapel Vestry some time ago placed some seats in the Whitechapel Road, outside the London Hospital, for the benefit of the people going there. So far good. But now comes the evil. These seats have been taken possession of by a class of "verminous persons," as they are called by Act of Parliament, who have neither home nor work, and they are occupied by them both day and night. It was stated at the Thames Police Court last week that sometimes as many as fifty persons slept there, and that the police could not keep them away, for as soon as their backs were turned they slept there again. The place, it was said, had become a rendezvous for thieves and tramps, and was a public danger. So difficult are the ways of philanthropy.

In the discussion on vaccination at Portsmouth it was held to be most important that the Vaccination Acts should contain some definition of what is meant by "successful vaccination." It was pointed out that the law acknowledges as successful vaccination a mark of any size, however microscopic, while the Local Government Board directs that there shall be four marks of a certain size; and it was very properly asked what is the position of a man who tries to explain that four marks are necessary when those to whom he is speaking know that a certificate of successful vaccination, which will be accepted as satisfying the Vaccination Acts, can be obtained, perhaps, not 50 yards off "for the modest sum of sixpence, and one pimple!" This is a real evil. Not only is it a perpetual incentive to bad vaccination, but it increases the number of those who, although vaccinated, are still susceptible, and thus piles up so-called "evidence" against vaccination for use by the anti-vaccinationists in years to come.

The *Medical and Surgical Review of Reviews* will in future be known as the *Medical Review*, which is an improvement. A cumbrous title is always an evil.

The International Ophthalmological Congress held during August at Utrecht was a great success. The discussions were animated, and the meetings well attended.

The question of the site of the Manchester Royal Infirmary is still unsettled. The war rages not only on the subject of terms between the authorities and the Corporation, but the whole question as to the removal of the hospital has arisen again.

The Medical Reform Association has made arrangements to hold a conference at St. Martin's Town Hall on October 10th and 11th. The subjects set down for discussion are "The Inquiry System," "Payments by Patients," and "Provident Dispensaries."

The most striking feature in the mortality returns for the past week is the high death-rate from diarrhœa in many of the large towns, which in Preston was equivalent to an annual rate of 16·4 per 1,000 living. In London, also, the diarrhœa rate was 201 above the corrected average, but that is a trifle compared with the rate in some of the provincial towns.

Dr. Hamilton Wright, of the Pathological Laboratory of the London County Council at Claybury, has been appointed by the Secretary of State for the Colonies to establish a pathological laboratory in the Malay Peninsular for the study of tropical diseases, and more especially of berri berri. Dr. Hamilton Wright's experience in the East and elsewhere especially fits him for his new post.

Reports of further cases of plague have been received during the week from Oporto and Alexandria, but in neither of these places does the disease appear to be showing much tendency to spread. The report from Astrakhan is to the effect that the first case occurred on July 23rd, and that since then there have been 24 cases, resulting in 23 deaths, and that the last case occurred on 21st ult.

We have on several occasions pointed out how serious would be the danger to the stability of the British Medical Association if the resolutions passed at the general meetings had any real force. The *Practitioner* writes in much the same vein. "It is of no particular consequence to anyone but themselves," it says, "that a small group of malcontents should hurl anathemas at the President and Council, and call for the punishment of an unoffending officer *pour encourager les autres*. It is of the greatest consequence to the Association, however, that a dozen members should be able, by a snatch vote, to appear to speak in the name of the British Medical Association in general meeting assembled. By a little skilful 'lobbying' it might be possible in this way to put the Association in the position of accepting homœopathy, or condemning vaccination. The 'general meeting' is doubtless useful as affording cranks and malcontents an opportunity of barking and biting, as 'tis their nature to, without doing serious mischief. But let it be clearly understood that the seat of authority in the Association is the Council, which alone can speak and act in its name. The resolutions of a 'general meeting' can no more bind the Council than those of a political meeting in St. James's Hall or in Hyde Park can bind the Government." This really just about expresses the true position of affairs.

